# The role of leptospiremia and specific immune response in severe leptospirosis

**DOI:** 10.1038/s41598-021-94073-z

**Published:** 2021-07-16

**Authors:** Umaporn Limothai, Nuttha Lumlertgul, Phatadon Sirivongrangson, Win Kulvichit, Sasipha Tachaboon, Janejira Dinhuzen, Watchadaporn Chaisuriyong, Sadudee Peerapornratana, Chintana Chirathaworn, Kearkiat Praditpornsilpa, Somchai Eiam-Ong, Kriang Tungsanga, Nattachai Srisawat

**Affiliations:** 1 Excellence Center for Critical Care Nephrology, Thai Red Cross Society, King Chulalongkorn Memorial Hospital, 1873, Rama 4 Rd., Lumphini, Pathumwan, Bangkok, 10330 Thailand; 2 Critical Care Nephrology Research Unit, Faculty of Medicine, Chulalongkorn University, Bangkok, Thailand; 3 Division of Nephrology, Department of Medicine, Faculty of Medicine, Chulalongkorn University, and King Chulalongkorn Memorial Hospital, Bangkok, Thailand; 4 Department of Laboratory Medicine, Faculty of Medicine, Chulalongkorn University, Bangkok, Thailand; 5 Department of Microbiology, Faculty of Medicine, Chulalongkorn University, Bangkok, Thailand; 6 Academy of Science, Royal Society of Thailand, Bangkok, Thailand; 7 Tropical Medicine Cluster, Chulalongkorn University, Bangkok, Thailand; 8 Excellence Center for Critical Care Medicine, King Chulalongkorn Memorial Hospital, Bangkok, Thailand; 9 Center for Critical Care Nephrology, The CRISMA Center, Department of Critical Care Medicine, University of Pittsburgh School of Medicine, Pittsburgh, PA USA

**Keywords:** Immunology, Microbiology, Molecular biology, Biomarkers, Diseases, Medical research, Molecular medicine, Pathogenesis, Risk factors

## Abstract

Leptospirosis can cause a high mortality rate, especially in severe cases. This multicenter cross-sectional study aimed to examine both host and pathogen factors that might contribute to the disease severity. A total of 217 leptospirosis patients were recruited and divided into two groups of non-severe and severe. Severe leptospirosis was defined by a modified sequential organ failure assessment (mSOFA) score of more than two or needed for mechanical ventilation support or had pulmonary hemorrhage or death. We found that leptospiremia, plasma neutrophil gelatinase-associated lipocalin (pNGAL), and interleukin 6 (IL-6) at the first day of enrollment (day 1) and microscopic agglutination test (MAT) titer at 7 days after enrollment (days 7) were significantly higher in the severe group than in the non-severe group. After adjustment for age, gender, and the days of fever, there were statistically significant associations of baseline leptospiremia level (OR 1.70, 95% CI 1.23–2.34, p = 0.001), pNGAL (OR 9.46, 95% CI 4.20–21.33, p < 0.001), and IL-6 (OR 2.82, 95% CI 1.96–4.07, p < 0.001) with the severity. In conclusion, a high leptospiremia, pNGAL, and IL-6 level at baseline were associated with severe leptospirosis.

## Introduction

Leptospirosis is one of the most important worldwide zoonosis and is a major public health issue in many countries. The disease is caused by spirochetes from the genus *Leptospira* and is transmitted by contact of abraded skin or mucous membranes with contaminated rodent urine, water, or soil^1^. There is an estimation of more than one million cases of severe leptospirosis per year worldwide^2^. Leptospirosis can cause severe multiple organ failure with a mortality rate as high as 50%^3–5^. Severe leptospirosis patients should receive early recognition and intensive medical care. The pathology of leptospirosis and the factors causing severe leptospirosis are still unclear.

Host and pathogen factors might play an important role in the pathogenesis of leptospirosis^1^. A high leptospiral load was found to be associated with the severity in several studies, but overall, the results are inconsistent^6–8^. Different *Leptospira* serogroups have different leptospiral lipopolysaccharides (LPS) and hemolysin, which are virulence factors and cause a difference in the disease severity^9,10^. However, the role of specific antibody response to pathogenic leptospires in the pathogenesis of severe leptospirosis is still unclear.

Host responses in leptospirosis are complex but important in the pathogenesis of the disease. Contact of the host with the pathogen causes the release of cytokines^11^. The extensive release of cytokines, including interleukin 6 (IL-6), interleukin 1 beta (IL-1β), and tumor necrosis factor-alpha (TNF-α), are known as a cytokine storm. Many studies have demonstrated the role of cytokines in the clinical manifestations and pathogenesis of leptospirosis. However, there is inconsistency among trials, probably due to heterogeneous study design^12–14^.

Renal biomarkers, such as neutrophil gelatinase-associated lipocalin (NGAL), might also be involved in the pathogenesis of leptospirosis. A previous study showed that plasma (p)NGAL and urine (u) NGAL were correlated with acute kidney injury (AKI) in patients with leptospirosis^15^. The NGAL is secreted by activated neutrophils in response to bacterial infections, but the roles of NGAL in the pathogenesis of leptospirosis are still unexplored^16^.

The results from previously mentioned studies showed that the pathology of leptospirosis remains unclear. This prospective cross-sectional study aimed to examine both host and pathogen factors that might be associated with the disease severity, including leptospiremia levels, a specific antibody response evaluated in terms of the microscopic agglutination test (MAT) titer, pNGAL, and IL-6.

## Methods

### Ethics statement

The study was conducted in compliance with the Declaration of Helsinki and the principles of Good Clinical Practice. All patients had given written informed consent. The study was approved by the Institutional Review Board of Faculty of Medicine, Chulalongkorn University, and the Institutional Review Board of Ministry of Public Health of Thailand (IRB no. 521/62).

### Study design and participant selection

This multicenter cross-sectional study carried out in a cohort of patients with leptospirosis was conducted in 15 hospitals in Sisaket province, Thailand, from November 2015 to December 2017. The list of the hospitals is provided in the acknowledgment section. Clinical suspicions for leptospirosis were a high fever (body temperature higher than 38 °C), severe myalgia, and history of exposure to reservoir animals or flood water. The inclusion criteria were (1) patients older than 18 years, (2) admitted in participating hospitals, and (3) patients were confirmed to have leptospirosis by being positive in one of the standard techniques (MAT, direct culture, and quantitative (q)PCR). We excluded the patients who suffered from other known infectious diseases. Blood samples were collected on the first day of enrollment (the first day of the clinical suspicious leptospirosis) and day 7 after enrollment. Samples were stored at − 80 °C until analyzed. All patients received a broad-spectrum antibiotic such 3rd generation cephalosporin and doxycycline within the first hour of recognizing leptospirosis according to the sepsis-3 guideline^17^.

### Outcomes

The outcome of interest was severe leptospirosis. We defined the patients as severe if the patient died, needed mechanical ventilation support, had pulmonary hemorrhage, or had at least one organ failure. We defined organ failure using a modified sequential organ failure assessment (mSOFA) score^18,19^ of more than two (which included coagulation, liver, cardiovascular and renal).

### Exposures

Exposures of interest were the level of leptospiremia, highest MAT titers, NGAL, and IL-6 level. Other covariates that might affect the outcome were sex, age, day of fever until enrolment, and antibiotic treatment.

The level of leptospiremia was measure by qPCR, as previously described^15,20^. Briefly, total DNA was extracted from 200 μl of the whole blood sample using a High Pure PCR Template Preparation kit (Roche Diagnostics, Germany) with 50 μl elution buffer. Pre-validated specific primers and TaqMan probe targeting lipL32 from Stoddard RA et al.^21^ were used in this study. The qPCR product size was 242 bp. The two primers used were as follows 45F primers (5′ AAG CAT TAC CGC TTG TGG TG3′) and 286R primers (5′ GAA CTC CCA TT T CAG CGA 3′) and Probe 189P (FAM-5′-AA AGC CAG GAC AAG CGC CG-3′-QSY). The qPCR reactions were performed in a final volume of 20 μl, corresponding to 5 μl of genomic DNA and 15 μl of reaction mix containing; the 10 μl SsoAdvanced Universal Probe Supermixs (Biorad Laboratories, USA) part number 64182275, providing final concentrations of 10 μM of each primer and 10 μM of the FAM-QSY labeled probe. A no-template control (NTC) that contained all the above reagents was also included to detect the presence of contaminating DNA. Amplification and fluorescence detection were conducted in the StepOnePlus Real-Time PCR Systems (Applied Biosystems, USA) with a program of 40 cycles, each cycle consisting of 95˚C for 15 s and 60 °C for one minute. The qPCR reactions were performed in duplicate. A negative result was assigned where no amplification occurred, i.e., the threshold cycle (Ct) value was greater than 40 cycles.

The MAT was performed as previously described^15,20^ using the standard protocol of the World Health Organization (WHO) guideline^22^. A positive MAT result defined as having a MAT titer of ≥ 1:400 in a single sample or four-fold rising in paired samples.

The pNGAL levels were measured by the quantikine human lipocalin-2/NGAL immunoassay (Catalog number: DLCN20, R&D Systems, Minneapolis, MN, USA) according to the manufacturer instructions.

The level of serum IL-6 was measured by a chemiluminescence method using the Elecsys IL-6 kit (Roche Diagnostics GmbH, Mannheim, Germany) with an analytical sensitivity of 1.5 pg/ml following the manufacturer's instructions.

### Direct culture of leptospires

We performed direct culture of leptospires by adding one drop of whole blood into 4 ml of liquid Ellinghausen–McCullough–Johnson–Harris (EMJH) medium and incubating this at 29 °C for 2 weeks. *Leptospira* were then observed and counted under dark-field microscopy by direct observation.

### Statistical analysis

Continuous variables are presented as the mean ± one standard deviation (SD) if parametric and as a median and interquartile range (IQR) in case of non-parametric data. Categorical variables are presented as numbers and percentages. Comparisons between groups were analyzed by the chi-square or Fisher’s exact test for categorical variables and by the Mann–Whitney U-test or Student’s t-test for quantitative variables. Logistic regression was used to assess the ORs relating to variables associated with severe leptospirosis. Additionally, we checked for multicollinearity in the regression models using the variance inflation factor (VIF), and no problem was detected. All statistical tests were two-sided, and p-values < 0.05 were considered significant. There were 16 samples on day 1 and 182 samples on day 7 after enrollment were missing values of leptospiremia due to the value below the limit of detection. While there were missing values of MAT titer due to the same issue in 207 samples on day 1 and 172 samples on day 7 after enrollment. Likewise, there were 22 missing values for NGAL and 30 missing values for IL-6. The value changes between two-time points (for the paired date on D1 and D7) within-group comparisons were tested by Wilcoxon signed-rank test. We performed all statistical analyses using the SPSS Version 22 software (SPSS, Chicago, IL), and figures were drawn using GraphPad Prism 9 (GraphPad Software Inc., California, USA).

## Results

### Participant characteristics

**Figure 1:**
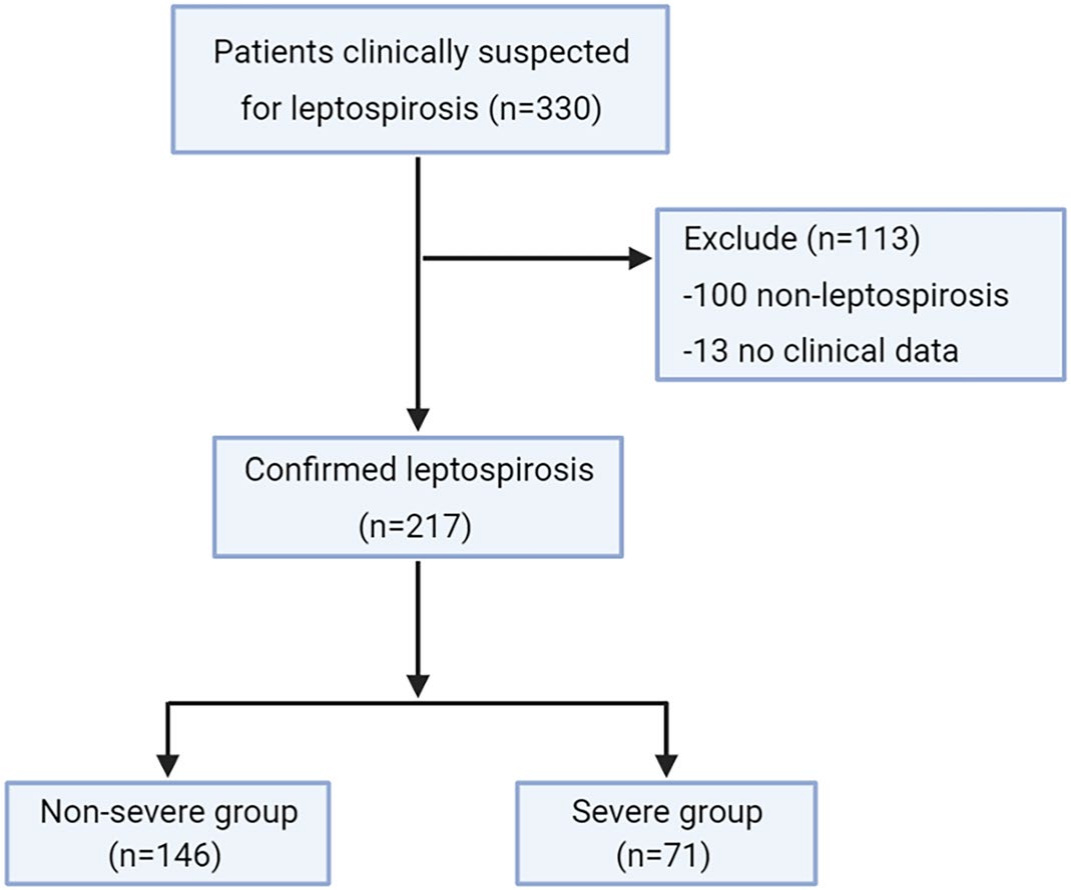
Study enrollment flow chart.

Among the 330 patients suspected of leptospirosis, those who were negative upon testing (non-leptospirosis patients) and patients with incomplete data were excluded leaving a total of 217 leptospirosis patients (146 patients in the non-severe group and 71 patients in the severe group) in this study (Fig. 1).

The characteristics of these patients are shown in Table 1. In total, 101 (69.2%) non-severe leptospirosis patients and 45 (30.8%) severe leptospirosis patients had a fever for 3 days or less before the diagnosis of leptospirosis infection. Compared with the non-severe group, the severe group had a significantly lower body temperature, systolic blood pressure, diastolic blood pressure, hemoglobin, platelet count, and serum bicarbonate, but a higher level of serum creatinine, white blood cell count, total bilirubin, direct bilirubin, and serum glutamic oxaloacetic transaminase. There was no significant difference in terms of the other clinical characteristics.

**Table 1:** Characteristics of leptospirosis patients.

Characteristic	Non-severe group (n = 146)	Severe group (n = 71)^a^	*p* value
Male gender, n (%)	121 (83.4)	59 (83.1)	0.9670
Age, years (mean, SD)	46.9 (17.0)	47.7 (16.2)	0.7330
**Fever days (median, IQR)**	2.5 (1.0, 4.0)	3.0 (2.0, 4.0)	0.2100
0–3 days, n (%)	101 (69.2)	45 (30.8)	
4 days or more, n (%)	41 (61.2)	26 (38.8)	
Body temperature (mean, SD)	38.4 (1.1)	37.8 (1.3)	0.001*
SBP, mmHg (median, IQR)	116.0 (101.3, 130.0)	104.0 (84.0, 120.0)	< 0.001*
DBP, mmHg (median, IQR)	68.0 (60.0, 76.0)	60.0 (52.0, 74.0)	0.001*
Creatinine, mg/dl (median, IQR)	1.1 (0.9, 1.3)	2.3 (1.1, 6.0)	< 0.001*
WBC × 10^3^/μl (median, IQR)	9.9 (6.9,12.9)	10.4 (7.0,13.9)	0.4240
Hb, g/dl (median, IQR)	12.5 (11.3, 13.8)	11.5 (9.5, 12.7)	< 0.001*
Platelet × 10^3^/μl (median, IQR)	155.0 (100.3, 214.0)	41.0 (25.0, 78.0)	< 0.001*
TB, mg/dl (median, IQR)	1.0 (0.7, 1.8)	2.5 (1.2, 7.9)	< 0.001*
DB, mg/dl (median, IQR)	0.5 (0.3, 0.8)	1.7 (0.6, 5.6)	< 0.001*
SGOT, u/l (median, IQR)	58.0 (34.8, 110.5)	96.0 (46.0, 170.0)	0.004*
SGPT, u/l (median, IQR)	52.0 (26.0, 84.5)	52.0 (31.0, 98.0)	0.3860
Na, mEq/l (median, IQR)	135.8 (132.0, 139.0)	134.2 (132.0, 138.0)	0.2050
K, mEq/l (median, IQR)	3.7 (3.3, 4.0)	3.6 (3.2, 4.1)	0.9050
HCO_3_^−^, mEq/l (median, IQR)	25.0 (23.0, 27.0)	20.2 (16.5, 24.0)	< 0.001*
Leptospiremia (copies/ml)	420.6 (154.2–1880.2)	1440.3 (172.0–7867.1)	0.009*
MAT titer	4000.0 (700.0–6400.0)	2000.0 (700.0–4000.0)	0.6140
pNGAL (ng/ml)	194.4 (75.0–350.5)	512.2 (224.3–968.0)	< 0.001*
IL-6 (pg/ml)	39.8 (13.6–145.4)	322.8 (54.2–4248.3)	< 0.001*

Data are shown as the mean (SD) or median (IQR).

*SBP* systolic blood pressure, *DBP* diastolic blood pressure, *WBC* white blood cells, *Hb* hemoglobin, *TB* total bilirubin, *DB* direct bilirubin, *SGOT* serum glutamic oxaloacetic transaminase, *SGPT* serum glutamate-pyruvate transaminase, *Na* sodium, *K* potassium, *HCO*_*3*_^*-*^ bicarbonate, *MAT* microscopic agglutination test, *NGAL* neutrophil gelatinase-associated lipocalin, *IL-6* interleukin 6.

*p-value < 0.05.

^a^Severe features were defined by one of the following criteria, 1. death, 2. requiring dialysis, or 3. organ failure. The organ failure was defined by an organ-specific Sequential Organ Failure Assessment (SOFA) score of more than 2.

The severe features of leptospirosis are summarized in Supplementary Table S1 online. Seven percent died, about 8% were diagnosed with severe liver failure, and 14% were diagnosed with severe renal failure. Approximately 5% needed dialysis, 21% had severe coagulopathy, and 7% had a severe cardiovascular system failure. The incidence of pulmonary hemorrhage was 8%. There were 12% of patients required mechanical ventilation.

### Distribution of *Leptospira* serogroups

**Figure 2:**
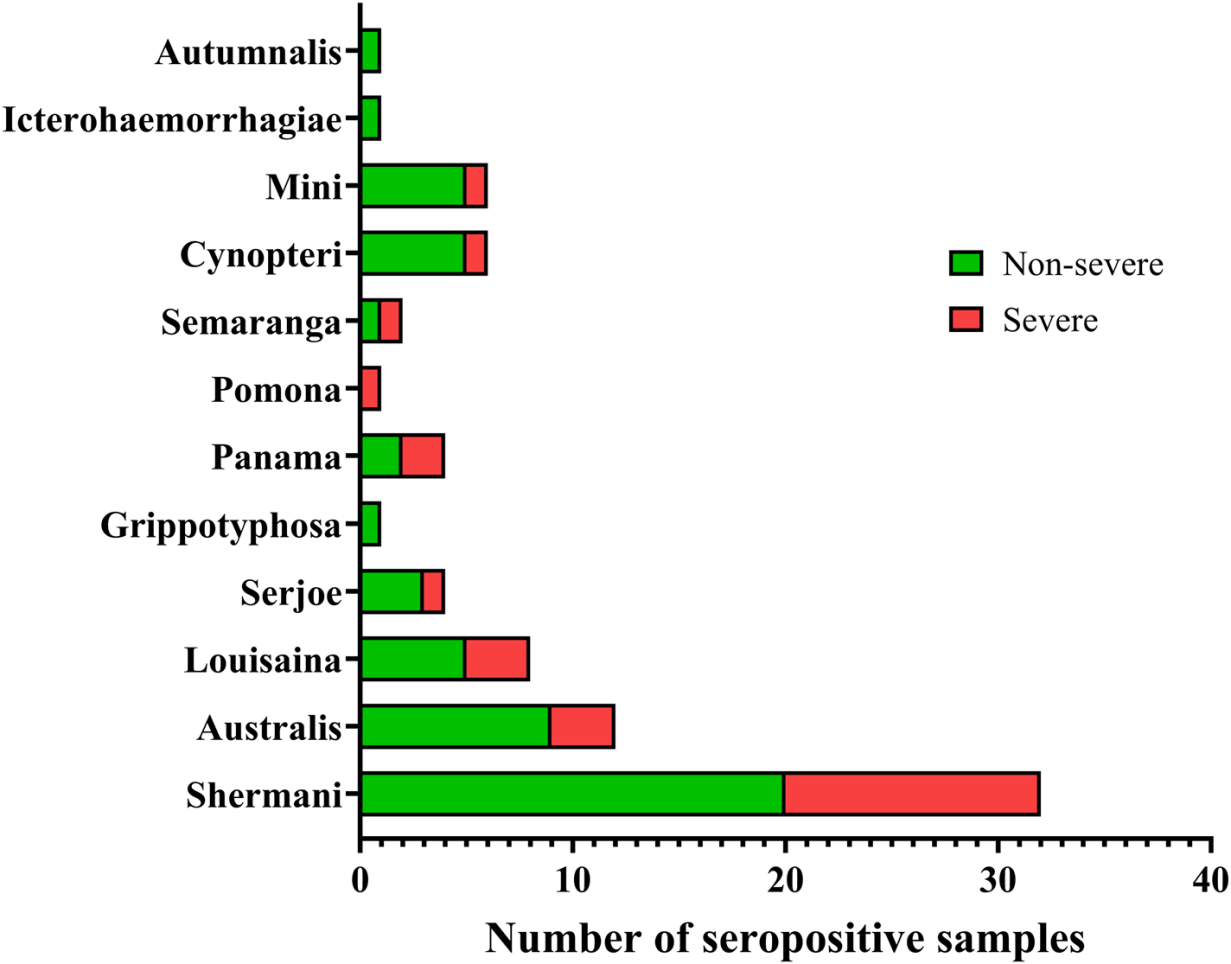
Seroprevalence of leptospirosis classified by severity.

Among the 217 study participants, 54 (24.9%) had *Leptospir*a agglutinating antibodies, as determined by the presence of MAT titer of ≥ 1:400 in a single sample or four-fold rise in paired samples. The most common serogroup was Shermani, followed by Australis and Louisaina. The serogroups of leptospirosis are summarized in Fig. 2.

### The level of leptospiremia, MAT titer, pNGAL, and IL-6 in relation to the severity of leptospirosis

**Figure 3:**
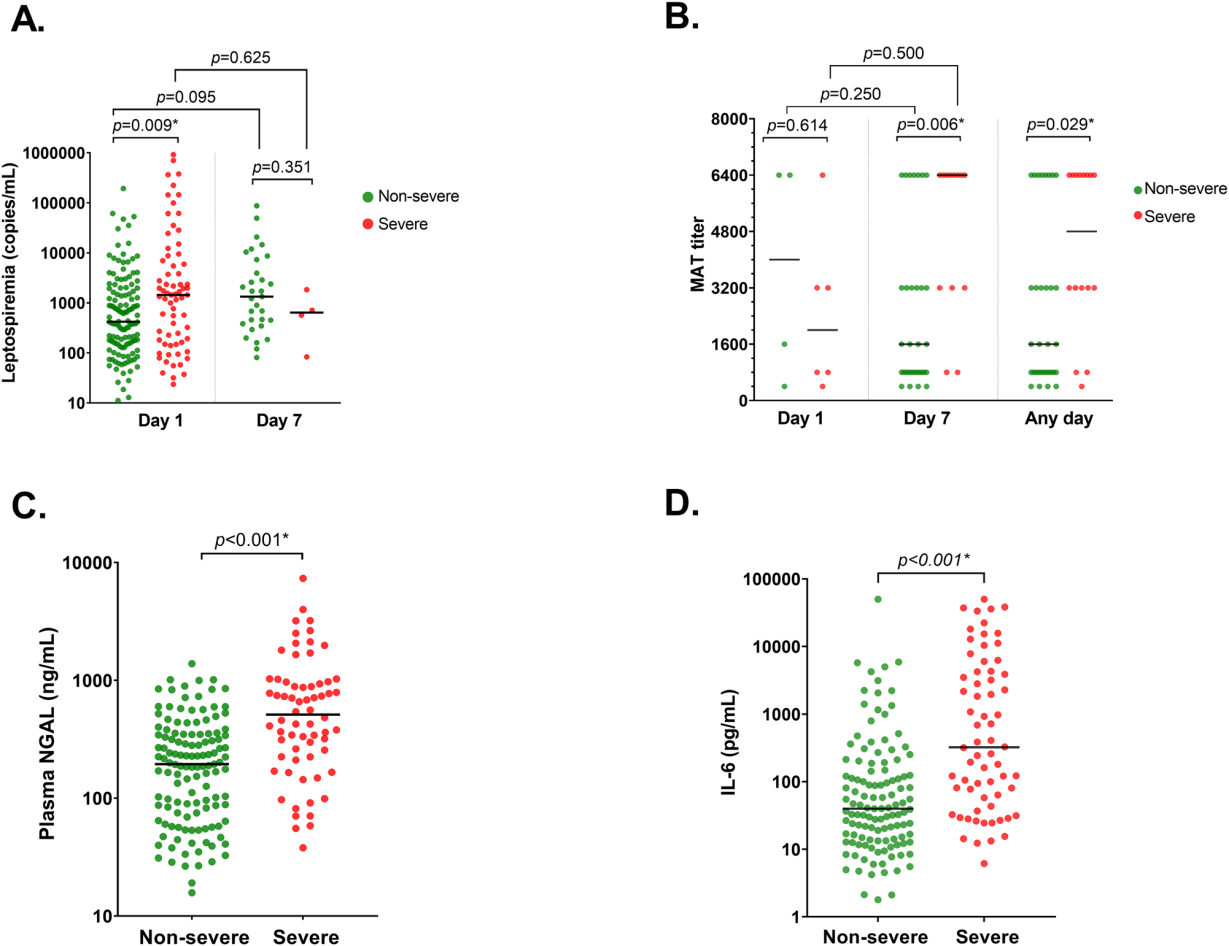
Level of all biomarkers in severe and non-severe leptospirosis patients, showing that for (**A**) Leptospiremia at the first day of enrolment and day 7 after enrolment, (**B**) Highest microscopic agglutination test (MAT) titers at the first day of enrolment and day 7 after enrolment and any day, (**C**) Plasma neutrophil gelatinase-associated lipocalin (NGAL) at the first day of enrolment, (**D**) Interleukin 6 (IL-6) at the first day of enrolment. Comparisons between groups were analyzed by the Mann–Whitney U-test. The value changes between two-time points (for the paired date on day 1 and day 7) within-group comparisons were tested by Wilcoxon signed-rank test.

On the first day of enrollment (day 1), the median level of leptospiremia was significantly higher in the severe group than the non-severe group (1440 copies/ml vs. 420 copies/ml), as shown in Fig. 3A. Moreover, the level of pNGAL (Fig. 3C) was also significantly higher in the severe group than in the non-severe group (512 ng/ml vs. 194 ng/ml). Compared with the non-severe group, the severe group also had a significantly higher IL-6 level (323 pg/ml vs. 40 pg/ml, Fig. 3D). *Leptospira* agglutinating antibodies were detected (MAT titer > 1:400) in only 10 (3.2%) patients and was included in the analysis. However, MAT titer did not show statistically significant differences between groups (Fig. 3B).

We also measure leptospiremia level and MAT titer 7 days after enrollment (day 7). Leptospiremia was detected in only 35 (11.0%) patients, and the level did not differ between severe and non-severe groups. Besides, *Leptospira* agglutinating antibodies were detected in 45 (14.2%) patients. Interestingly, the maximum MAT titer at day 7 (Fig. 3B) was significantly higher in the severe group (6400 vs. 1600) than in the non-severe group. Similarly, the maximum MAT titer at any day (combined both day 1 and day 7) was higher in the severe group (4800 vs. 1600) than in the non-severe group.

The ROC receiver operating characteristic (ROC) curve analyses were performed to evaluate the association between biomarkers at baseline and severe leptospirosis. The results indicated that the pNGAL and IL-6 levels at baseline showed the best diagnostic performance with an equal area under the ROC (AUC) as 0.75. The derived sensitivities and specificities for a cutoff of each biomarker to provide the maximum summation of the sensitivity and specificity are shown in Table 2.

**Table 2:** Area under the receiver operating curve for the association between baseline biomarkers and severe leptospirosis.

Parameters	AUC ROC	SE	p value	95% CI	Cut-off	Sensitivity	Specificity
Leptospiremia	0.61	0.04	0.010*	0.52–0.70	953	0.59	0.67
MAT titer	0.40	0.20	0.594	0.00–0.80	600	0.83	0.25
NGAL	0.75	0.04	< 0.001	0.67–0.82	360	0.62	0.78
IL-6	0.75	0.04	< 0.001	0.67–0.82	121	0.64	0.74

*AUC/ROC* area under curve of receiver operating curve, *SE* standard error, *CI* confidence interval, *Sens* sensitivity, *Spec* specificity, *pNGAL* plasma neutrophil gelatinase-associated lipocalin, *IL-6* interleukin 6, *MAT* microscopic agglutination test.

### Regression analysis of factors associated with severe leptospirosis

Based on logistic regression analyses, parameters were further investigated to identify variables associated with severe leptospirosis (Table 3). From the univariable regression analysis, the factors associated with severe leptospirosis were leptospiremia, pNGAL, IL-6 levels at baseline (day 1).

**Table 3:** Regression analysis of factors associated with severe leptospirosis.

Parameters	Odds ratio (95% CI) unadjusted	p-value	Odds ratio (95% CI) Adjusted^a^	p-value
Gender	1.02 (0.48–2.16)	0.967	1.00 (0.99–1.02)	0.807
Age	1.00 (0.99–1.02)	0.731	0.96 (0.44–2.11)	0.925
Days of fever until enrollment	1.05 (0.92–1.21)	0.456	1.05 (0.92–1.21)	0.465
Leptospiremia (log_10_ copies/ml)	1.66 (1.21–2.27)	0.002*	1.70 (1.23–2.34)	0.001*
MAT titer (log scale)	0.49 (0.03–8.11)	0.617	0.35 (0.01–8.79)	0.523
Plasma NGAL (log_10_ ng/ml)	8.51 (3.88–18.67)	< 0.001*	9.46 (4.20–21.33)	< 0.001*
IL-6 (log_10_ pg/ml)	2.61 (1.84–3.70)	< 0.001*	2.82 (1.96–4.07)	< 0.001*

*pNGAL* plasma neutrophil gelatinase-associated lipocalin, *IL-6* interleukin 6, *MAT* microscopic agglutination test.

*p-value < 0.05.

^a^Adjusted for gender, age, and days of fever until enrollment.

After adjusted the analysis of biomarkers by gender, age, and days of fever until enrollment, the multivariate regression analysis shown that high leptospiremia, pNGAL, and IL-6 levels at baseline (day 1) were factors associated with severe leptospirosis.

### Biomarkers associated with the type of organ failure in severe leptospirosis

We further explored the associations of each biomarker and types of organ failure, as shown in Table 4. The result indicated that leptospiremia, pNGAL, and IL-6 levels at baseline (day 1) were associated with severe coagulopathy, severe cardiovascular system failure, pulmonary hemorrhage, and severe respiratory failure. The pNGAL and IL-6 were also associated with severe renal failure and severe liver failure. Besides, the MAT titers 7 days after enrollment were associated with severe coagulopathy and pulmonary hemorrhage.

**Table 4:** Association between biomarkers and severe leptospirosis by type of organ failure (represented by *p*-value).

Parameters	Severe coagulopathy	Severe renal failure	Severe liver failure	Severe cardiovascular system failure	Pulmonary hemorrhage	Severe respiratory failure^a^
Leptospiremia at day 1	0.003*	0.215	0.129	0.009*	0.001*	0.013*
Leptospiremia at day 7	0.421	0.843	0.871	0.692	NA	0.943
MAT titer at day 1	0.127	0.907	0.722	0.286	0.286	0.477
MAT titer at day 7	0.032*	0.803	0.327	0.087	0.034*	0.231
MAT titer at any day	0.121	0.716	0.313	0.084	0.033*	0.227
Plasma NGAL at day 1	0.002*	< 0.001	< 0.001	< 0.001	< 0.001	< 0.001
IL-6 at day 1	0.003*	< 0.001	< 0.001	< 0.001	< 0.001	< 0.001

*pNGAL* plasma neutrophil gelatinase-associated lipocalin, *IL-6* interleukin 6, *MAT* microscopic agglutination test, *NA* not available.

*p-value < 0.05.

^a^Severe respiratory failure was defined as requiring mechanical ventilation. The data were expressed as a *p*-value from the Mann–Whitney U test.

## Discussion

Pathogenesis of severe leptospirosis involves many complex mechanisms, including direct leptospiral invasion, the indirect effect of immune responses, cellular cytokines, and humoral immune responses^11^. In this study, we explored the role of cellular immune responses by measuring the serum IL-6 and humoral immune responses by measuring the MAT titer. Moreover, renal biomarkers (pNGAL) and leptospiremia levels were also examined. The results revealed that the level of leptospiremia, pNGAL, and IL-6 at baseline (day 1) were significantly higher in the severe group than the non-severe group. Moreover, high MAT titer at 7 days after enrollment was also associated with severe leptospirosis.

Quantitative leptospiremia by qPCR could provide an accurate and timely diagnosis for leptospirosis at the point of care^23^. However, due to its higher cost (than other diagnostic methods) and logistical challenges, it has not been widely used as an early diagnostic tool presently. This study showed increased leptospiremia levels in the severe leptospirosis group compared to the non-severe group on the first day of enrollment. These results were well correlated with a previous study conducted in Martinique to identify factors associated with disease severity, where the leptospiremia level was significantly higher among patients with severe disease^7^. Another case–control study in New Caledonia found that leptospiremia with > 1000 leptospires/ml was associated with severe leptospirosis^8^. It is possible that patients with a high serum leptospiral load have a higher disease burden and so greater disease severity. However, we found that the association between leptospiromia level and severe leptospirosis was disappeared 7 days after enrollment. A study from Sri Lanka showed that the quantitative leptospiremia level was not correlated with the clinical manifestations and outcome of leptospirosis^6^. In fact, the results from the previously mentioned studies were conflicting. So they must be interpreted with caution due to each study using different criteria to determine disease severity. Moreover, there were differences in the sample collection time-point among these studies, which might affect the results.

Traditionally, the MAT is still being used as the gold standard for leptospirosis diagnosis in most countries. The present study is among the first to investigate MAT titer as a potential severity marker. We found a significant association between a high MAT titer, a method for the quantitative detection of antibodies, and severe leptospirosis 7 days after enrollment. However, the association did not observe on the first day of enrollment, which may be due to the MAT titer being undetectable in most cases. Currently, it is unclear whether the MAT titer level is associated with severe leptospirosis. It is possible that a reactive MAT titer in a patient with a history of previous leptospirosis may be caused by the anamnestic response, which causes the MAT titer to rise to the previous serogroup instead of the current one^24^. Since Thailand is one of the endemic areas of leptospirosis, reinfection with a different serogroup is possible^25^. Therefore, patients with non-protective antibodies might still have a severe disease even in the presence of a high MAT titer. This would explain our results that a high MAT titer is not a protective factor against severe disease. In contrast, a recent study from Brazil showed that fatal leptospirosis cases had higher bacterial loads and lower anti-Leptospira antibody titers^26^. However, the results must be interpreted with caution due to the small sample size.

The pNGAL is known to be renal biomarkers that can predict AKI. Stressed kidney epithelial cells secrete NGAL in response to the various causes of injury. This study showed that pNGAL was associated with severe leptospirosis and severe renal failure. Previous studies showed that sepsis was associated with an elevated pNGAL level independent of the renal impairment degree^27^, and pNGAL as a biomarker could be used as a predictor of the severity and 28-day mortality in severe sepsis^28^. The use of pNGAL as a biomarker still has an advantage in the setting of anuria patients. Also, we found that pNGAL was associated with severe coagulopathy. To date, there are limited data on the relationship between NGAL and coagulation. A previous study reported that patients with disseminated intravascular coagulation (DIC) who had a DIC score of five or more had higher serum NGAL levels than those who had not^29^. However, the mechanism to explain the association between NGAL and coagulation dysfunction is still unclear, but it was proposed that DIC might cause advanced coagulation activation that then induced a high plasma NGAL level.

Our study also shows a correlation between serum IL-6 levels and severe leptospirosis. IL-6 is a multifunctional cytokine that plays a critical role in the host defense mechanism^30^. Several studies have demonstrated that the serum concentrations of IL-6 were significantly higher in severe leptospirosis patients than non-severe patients^14,31,32^. The previous studies also showed a significantly higher IL-6 expression in fatal cases when compared with mild cases of leptospirosis^14,31^. Interestingly, Papa A. and Kotrotsiou T. reported an increased IL-6 level at the early stages (1–10 days post-onset of illness) and declined in the late phase of leptospirosis infection (11–15 days post-onset of illness)^33^. Correspondingly, Chirathaworn et al. showed that IL-6 level was increased following acute leptospirosis infection and decreased in the convalescent phase. In the acute phase, IL-6 also correlated with organ dysfunction; however, such a correlation disappeared in the convalescent phase^32^. Therefore, the specimen collection time could affect the significant levels, particularly as biomarkers for disease severity monitoring.

Our study has several strengths. Firstly, to our knowledge, this study used one of the largest multicenter leptospirosis cohorts with over 200 participants. Secondly, this study used organ-specific SOFA scores to diagnose severe leptospirosis. Indeed, this data should be interpreted with caution because it was different from previous studies that used various heterogeneous criteria to diagnose severe leptospirosis. We believe that the SOFA score is more suitable because it is standard and universally used in current trials of critical care. Thirdly, we have leptospiral load and MAT titer at different times (day 1 and days 7), which would help better understand the disease dynamics. We also took a look at potential confounding factors that might affect the outcome, including sex, age, day of fever until enrolment, and antibiotic treatment. There was no significant difference in terms of gender, age, and day of fever until enrolment in this cohort. In addition, antibiotic therapy was standardized in this study. Finally, we used the adjusted model with days of fever to reduce the bias from collecting specimens on different days post initial infection, which differs from other studies.

This study is not without limitations. Firstly, due to the study design, the blood tests were unable to be obtained before hospitalization or at the disease's onset. To reduce this bias, we adjusted the analysis of biomarkers with the day of fever. Secondly, we measure pNGAL and IL-6 only on the first day of clinical suspicion of having leptospirosis. However, one laboratory testing at the hospital was pragmatic in clinical practice, and our data can be applied in this setting. Thirdly, the organ-specific SOFA scores were calculated using routine biomarkers such as bilirubin, creatinine, and platelet. Unfortunately, we could not analyze these routine biomarkers as covariables. Lastly, the biomarkers and laboratory methods we used are not commonly available outside tertiary care hospitals in Thailand. The use of NGAL, cytokines and leptospiral load as markers is still not available in most parts of Thailand, in common with some other countries in endemic areas. Nonetheless, the benefit of the investigation in this study had been demonstrated. It could help to improve the understanding of the pathophysiology as well as determine future research directions.

In summary, our data demonstrated that a high leptospiremia, pNGAL, and IL-6 level at baseline were associated with severe leptospirosis.

## Supplementary Information

Supplementary Information.
