# Supplementary material for: The role of leptospiremia and specific immune response in severe leptospirosis
Source: Sci Rep. 2021 Jul 16;11:14630. doi: 10.1038/s41598-021-94073-z (PMC8285422; doi:10.1038/s41598-021-94073-z)
Supplement: Supplementary file 1 — Supplementary Information. [file 41598_2021_94073_MOESM1_ESM.docx]

# **The role of leptospiremia and specific immune response in severe leptospirosis.**

Umaporn Limothai^1,2*^, Nuttha Lumlertgul^1,2,3*^, Phatadon Sirivongrangson^1,2*^, Win Kulvichit^1,2^, Sasipha Tachaboon^1,2^, Janejira Dinhuzen^1,2^, Watchadaporn Chaisuriyong^1,2^, Sadudee Peerapornratana^1,2,3,4^, Chintana Chirathaworn^5^, Kearkiat Praditpornsilpa^3^, Somchai Eiam-Ong^3^, Kriang Tungsanga^3^, Nattachai Srisawat^1,2,3,6,7,8,9**^

**Supplementary Table S1.** Characteristics of severe leptospirosis patients*

| **Characteristic** | **N (%)** |
| --- | --- |
| Dead | 15 (6.9) |
| Severe liver failure | 18 (8.3) |
| Severe renal failure | 30 (13.8) |
| Received dialysis | 10 (4.6) |
| Severe coagulopathy | 45 (20.7) |
| Severe cardiovascular system failure | 15 (6.9) |
| Pulmonary hemorrhage | 18 (8.3) |
| Severe respiratory failure** | 27 (12.4) |

]

*Severe features were defined by one of the following criteria, 1. death, 2. requiring dialysis, or 3. organ failure. The organ failure was defined by an organ-specific Sequential Organ Failure Assessment (SOFA) score of more than 2. **Severe respiratory failure was defined as requiring mechanical ventilation.
